# A structured professional development curriculum for postdoctoral fellows leads to recognized knowledge growth

**DOI:** 10.1371/journal.pone.0260212

**Published:** 2021-11-22

**Authors:** Kaylee Steen, Jay Vornhagen, Zara Y. Weinberg, Julie Boulanger-Bertolus, Arvind Rao, Margery Evans Gardner, Shoba Subramanian

**Affiliations:** 1 Cell & Developmental Biology Department, University of Michigan Medical School, Ann Arbor, Michigan, United States of America; 2 Department of Pathology, University of Michigan Medical School, Ann Arbor, MI, United States of America; 3 Department of Pharmacology, University of Michigan Medical School, Ann Arbor, MI, United States of America; 4 Department of Anesthesiology, Center for Consciousness Science, University of Michigan, Ann Arbor, MI, United States of America; 5 Departments of Computational Medicine and Bioinformatics, Radiation Oncology, MIDAS, Ann Arbor, United States of America; 6 Office of Graduate and Postdoctoral Studies, University of Michigan Medical School, Ann Arbor, MI, United States of America; 7 Department of Cell and Developmental Biology, Office of Graduate and Postdoctoral Studies, University of Michigan Medical School, Ann Arbor, MI, United States of America; Rush University Medical Center, UNITED STATES

## Abstract

Postdoctoral training enables research independence and professional readiness. National reports have emphasized professional development as a critical component of this training period. In response, many institutions are establishing transferable skills training workshops for postdocs; however, the lack of structured programs and an absence of methods to assess outcomes beyond participant satisfaction surveys are critical gaps in postdoctoral training. To address these shortcomings, we took the approach of structured programming and developed a method for controlled assessment of outcomes. Our program You^3^ (You, Your Team, Your Project), co-designed by postdoctoral fellows, focused on discussing specific management and leadership skills agnostic of ultimate career path(s) in a structured manner. We then measured outcomes in a controlled manner, by systematically comparing perceived knowledge and growth as indicators of awareness and confidence in participants against that of non-participants as the control group. You^3^ participants self-rated greater growth in targeted competencies compared to non-participants independent of the number of years of training. This growth was shown by multiple criteria including self-reporting and associative analysis. Correspondingly, You^3^ participants reported greater knowledge in 75% of the modules when compared to controls. These data indicate that structured learning, where postdocs commit to a curriculum via a cohort-structure, leads to positive outcomes and provides a framework for programs to assess outcomes in a rigorous manner.

## Introduction

Postdoctoral training is a unique period that is defined by the National Postdoctoral Association (nationalpostdoc.org) as “temporary time for scholarly work as well as acquisition of professional skills for career success”. To be successful in their next career step, postdoctoral fellows (referred to as ‘postdocs’ for the remainder of this article) need to develop a wide variety of skills, including technical and non-technical skills. Although the importance of technical skills has been recognized for many years, growing evidence shows non-technical skills, also known as transferable skills, are similarly vital to succeeding as a professional during and beyond training [1–5]. This importance of transferable skill training is also underscored by a study conducted in 2016, which identified major gaps between the labor market and skills in the biomedical research workforce [6]. Therefore, a well-rounded portfolio is especially important in current times, where less than 25% of the PhD graduates move on to a tenure track (TT) position, while the rest pursue a distinct array of individual paths [7].

The unique nature of the postdoctoral training period presents several challenges for developing transferable skills. Postdocs are hired by individual faculty advisors and do not typically enter the academic system as part of a cohort. They start their appointments asynchronously, and the orientation, onboarding routines, and access to non-research resources are highly variable within and across academic units. Postdoctoral training is largely apprentice-based, leading to disparities in the scope and breadth of training, depending on their advisor’s experience and encouragement in combination with institutional support for expanded learning opportunities. The lack of structured professional skill development opportunities underserve the nearly 80% of PhD students in biomedicine that go on to do postdoctoral training [8]. Many institutions face these limitations [9], due to a combination of constraints on incentives, resources, personnel, and budget. Even in the case where there are more professional development resources to build programs, both access to and utilization of these programs vary heavily depending on the individual trainee’s circumstances.

Two key factors that are critical limitations in training postdocs are a lack of structured programs for standardized training and a lack of methods to assess outcomes scientifically. Research institutions are investing resources at the university, college, and departmental levels to provide seminars and workshops often based on the postdoc core competency guidelines set by the National Postdoctoral Association. Although well intentioned, these programs often follow an *ad hoc* pattern of topics and schedules with little structure, accountability, or evaluation of efficacy. A lack of a sequential structure leads to discontinuous learning, which can be ineffective [10]. Moreover, the absence of a cohort or peer structure for postdocs compounds shortcomings within these programs, leading to loss of peer-community groups and peer learning with a higher impact on historically excluded groups [11, 12]. Often, professional development programs are segregated by academic or non-academic career paths. This segregation makes it difficult for those who are undecided to choose the right program for them and might inadvertently make it difficult for postdocs to navigate non-academic career plans without fear or judgement. These career-goal based professional development programs also lose the benefit of exchange and discourse between individuals of different mindsets and backgrounds. The lack of structure is beginning to be addressed by systems like the NIH BEST program [13] which recognizes the vital role of a robust infrastructure for professional development in scientific and science-related careers. However, even when institutes put in place structured professional development programs, outcomes are primarily assessed using satisfaction surveys. A lack of rigorous assessments for professional development program efficacy is pervasive across all types of skill building programs partly because of the lack of useful mechanism(s) for conventional formative or summative assessments. This gap is also driven by the non-technical nature of the topics covered, which are challenging to quantify in advanced learners. As a result, we have no clear and controlled assessment methods that measure impact via participants’ knowledge and growth in the topic in a systematic manner.

To effectively address these key limitations, we developed and implemented an innovative leadership and management program titled “You^3”^. The goal of our structured program, co-designed and co-created by four postdoctoral fellows, was to implement a cohort-based and sequential curriculum, and to measure its impact by comparing perceived growth and knowledge as metrics of awareness and confidence in participants against that of a control group of non-participants. Our results show that participants increased their awareness and confidence in key transferable skills, when compared to the control population who did not participate in our program. We propose You^3^ as a scalable framework that can seed and establish similar opportunities for postdocs in other institutions across the nation.

## Materials and methods

### Needs assessment data collection

#### Employer survey

The Office of Graduate and Postdoctoral Studies had commissioned miLEAD, a trainee-run non-profit consulting group on campus, to better understand career preparation and job market trends for graduate and postdoc learners. In their report, the miLEAD group examined Ph.D. training and career outcomes from 1974 to 2015, including the U-M Rackham Graduate School 2016 Ph.D. Program Statistics, exit surveys from several schools, and available national surveys. Based on these combined data, miLEAD defined nine career sectors (Academia, National Labs, Pharma, Biotech, IP & Patent Law, Policy, Scientific Writing, Consulting, and Data Science) of focus. They interviewed 36 hiring recruiters and analyzed 192 PhD entry level job postings from these sectors. miLEAD identified the top four professional skills based on the frequency mentioned and essentiality of each skill. These skills were then organized by perceived strengths and weaknesses hiring managers described of Ph.D. holding candidates for these skills. miLEAD also identified the training needs of postdocs through a series of focus groups (n = 18) and surveys (n = 237) sent to University of Michigan Medical School (UMMS) postdocs. The postdoc needs data are represented via a summary of miLEAD group’s key findings.

#### UMMS Faculty survey

Before program planning, we sent an anonymous needs assessment survey to faculty at the University of Michigan Medical School. The survey asked faculty to select all skills that applied to the question “Which transferable skills do you wish you had developed further prior to becoming a faculty member?”. Of the 266 respondents, 32.7% were Professors, 26.6% were Assistant Professors, 21.4% were Associate Professors, 5.2% were Clinical Instructional Faculty, 3% were Lecturers, 1.5% were Instructors, and 9.3% were Other. Faculty were able to select more than one skill in their response, and we counted the total number of times each topic was selected across each faculty category.

### You^3^ participant enrollment

Advertisement material of the You^3^ program was widely distributed to all UMMS postdoctoral fellows via emails and flyers. Prospective applicants were asked to reflect on their professional experience and goals in order to assess their intentions and commitment to the You^3^ program. To avoid attracting postdoctoral fellows that were interested in a specific career trajectory, the questions were intentionally broad. Applicants were also asked their career interest and were able to select as many options as were applicable. S1 Fig displays complete application requirements. Applicants were then randomly divided into two groups, and each applicant group was evaluated by three members of the You^3^ steering committee. For reviewing, we utilized a scale of 1–5 with 5 being excellent alignment between the applicant’s goals and the learning goals of You^3^ and 1 being low alignment. Applicants with an average score of 4 or higher were automatically accepted; applicants with a score between 3 and 4 were discussed by the committee, and a select few were requested to interview to better gauge their overall understanding of, interest in, and commitment to the program. By this process, 33 of 35 applications were sent an acceptance letter into the program. 32 accepted the offer and committed to participation. Relevant data sets are provided in supporting files.

### Self-assessment pre-course survey to You^3^ participants

Prior to starting the You^3^ program, participants were surveyed on their training history in the core competencies of the program. Their responses were categorized into formal training (led by an instructor with engagement activities, i.e. workshop, class), informal training (purely self-driven, i.e. online webinar, reading, etc.), and no training prior to enrollment.

### Self-assessment outcomes survey to control and participant groups

All UMMS postdoctoral fellows received an online Qualtrics survey (IRB Exempt #HUM00172637) to anonymously assess their self-reported knowledge and growth in the eight topics covered by the You^3^ program (S1 Appendix). We followed University of Michigan’s recommendation on consent for studies deemed exempt from IRB. A detailed informed consent information document was shared with the email invitation to postdocs to participate in the anonymous survey. Our survey did not collect any identifiable information and participation was voluntary. The digital informed consent statement is available in S1 Appendix. Based on each participant’s response, the survey items were binned according to the trainee’s prior experience with any University of Michigan skills building workshop, no skills building workshop experience, or participation in the You^3^ program. The following distribution of survey participants completed the questionnaire: You^3^ participants (n = 23); control group who participated in some kind of professional development programming at UM (n = 18); and control group that did not participate in relevant programming (n = 35).

#### Statistical analysis for self-reported knowledge and growth metrics

For knowledge metric analysis, confidence score values were compared using Kruskal-Wallis test followed by Dunn’s posthoc test, and Pearson correlation coefficients and P values were calculated for each category and metric. For growth metric analysis, text-based respondent data was converted into numerical data, wherein 4 categories (no growth, low growth, medium growth, high growth) were converted to numerical values (1–4, respectively). Odds ratios and associated 95% confidence intervals (Bland-Altman method) [14], as well as Pearson correlation coefficients and P values, were calculated for each category and metric. For reliability analysis, Cronbach’s alpha was calculated for each question block (Growth and Current Knowledge) in R version 4.0.2 using the “psych” package. Kruskal-Wallis test followed by Dunn’s posthoc test were performed using GraphPad Prism version 8, and Pearson correlation coefficient and P-value calculations were performed and visualized in R version 4.0.2 using the “Hmisc” package. Relevant data sets are provided in supporting files.

## Results

To establish a comprehensive program, we focused on four achievable goals: (a) addressing specific needs of postdocs immediately transitioning into the workforce, (b) using a holistic view of professional development to deliver training over eight consecutive weeks, (c) developing a cohort model toward fostering community, and (d) enabling management and leadership skills applicable across diverse career pathways. At the end of the program, we developed an instrument to measure the impact of our program in a controlled manner by comparing You^3^ participants’ self-reported knowledge and perceived value calibrated by growth in You^3^ topic areas compared to non-participant controls.

We used evidence-based data to determine the core topic list. We factored in needs assessments and workforce requirements from the perspective of three key stakeholder groups: a) employers hiring PhD holders, b) UM Medical School (UMMS) faculty, and c) postdocs at our school during the time of survey.

**Fig 1:**
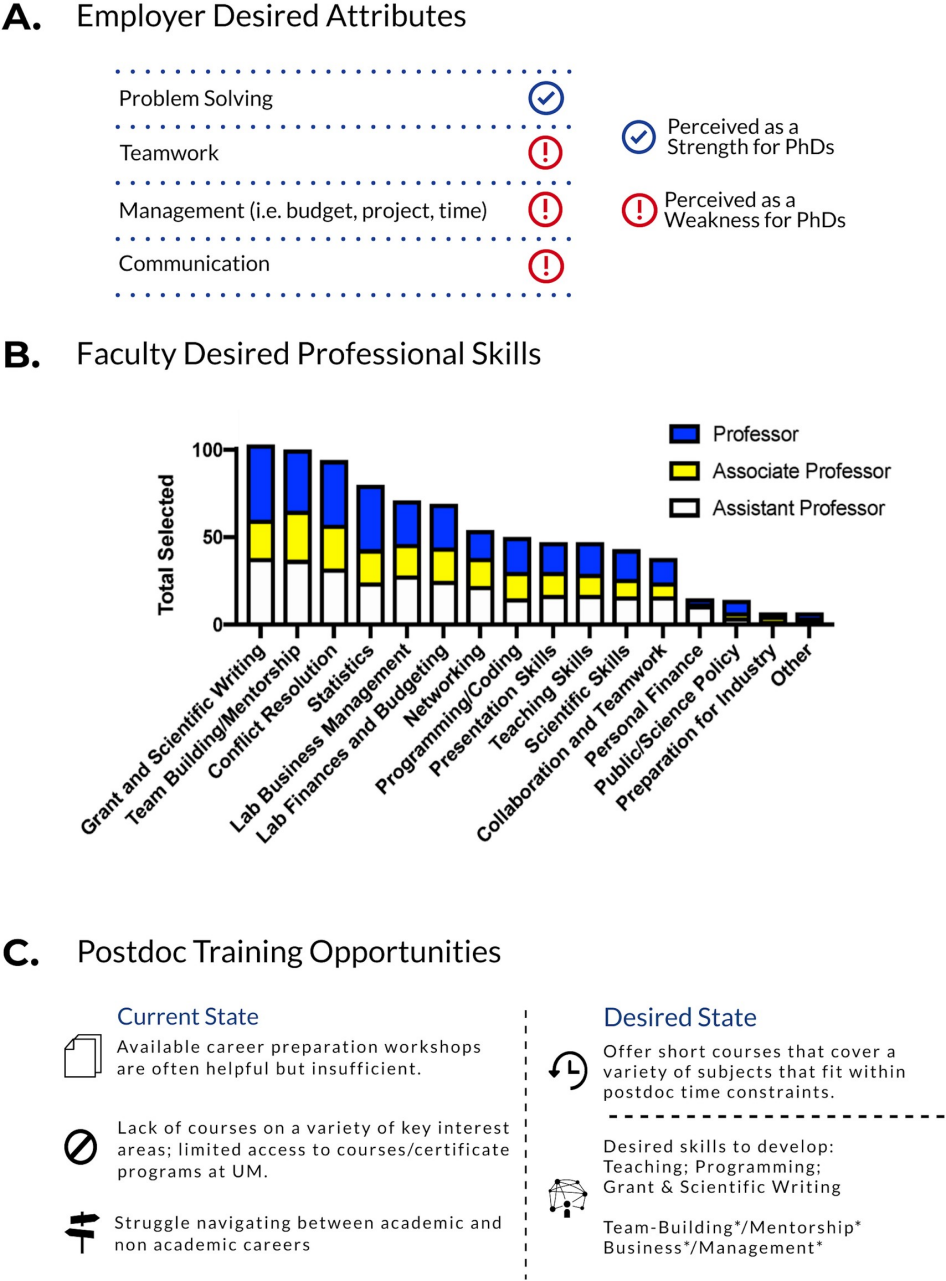
Postdocs do not receive adequate professional training based on needs assessment surveys and interviews. (A) miLEAD Consulting Group interviewed 36 industry professionals and analyzed 192 non-academic job postings to identify the professional skills employers seek in applicants. The top soft skills attributes desired by hiring managers were marked as perceived weaknesses and strengths of PhDs based on industry interviews. (B) A needs assessment survey was distributed to UMMS faculty (266 responded), who rated the value each skill contributed to their career success. For clarity, shown are responses from full, associate, and assistant professors, but the results were consistent across all faculty categories. (C) miLEAD Consulting Group surveyed and interviewed current and former postdocs from 14 departments within UMMS in order to determine the professional development training postdocs receive. Their relevant responses are summarized as current and desired state of professional development training. The most desired professional skills that are career agnostic is indicated by asterisks.

First, we focused on the needs of employers by identifying deficiencies in professional skills for PhD job applicants based on interviews with 36 industry professionals and analysis of 192 non-academic job postings (data collected and analyzed by miLEAD Consulting Group, Fig 1A). From the top skills that emerged, communication, teamwork and management (project, time, personnel, and budgeting) were perceived as weaknesses of PhD degree holders seeking employment (Fig 1A). Next, to understand needs of academic faculty jobs, an anonymous survey was distributed to UMMS faculty independent of the miLEAD data. In this survey, we asked current faculty, across all ranks in our college to identify one or more of 15 transferable skills that they were not proficient in prior to becoming a faculty member. Data from the 266 responses pointed at many common skills that were identified as deficiencies prior to assuming their faculty roles. Starting with the most frequently indicated, these skills included Grant/Scientific Writing, Team Building/Mentorship, Conflict Resolution, Statistics, Lab Business Management, and Lab Finances/Budgeting (Fig 1B). These data include responses from Assistant, Associate and Full Professors, and the results are consistent between all tracks. Finally, we compared the skills needed for successful careers to the gaps in training identified by current postdocs. Based on interviews and surveys collected from miLEAD Consulting, the current state of training was not deemed sufficient in preparing postdocs for professional careers, and the desired state is summarized in Fig 1C. These collective data from trainees, employers, and faculty are consistent with published work on PhD-holding employees [5].

**Fig 2:**
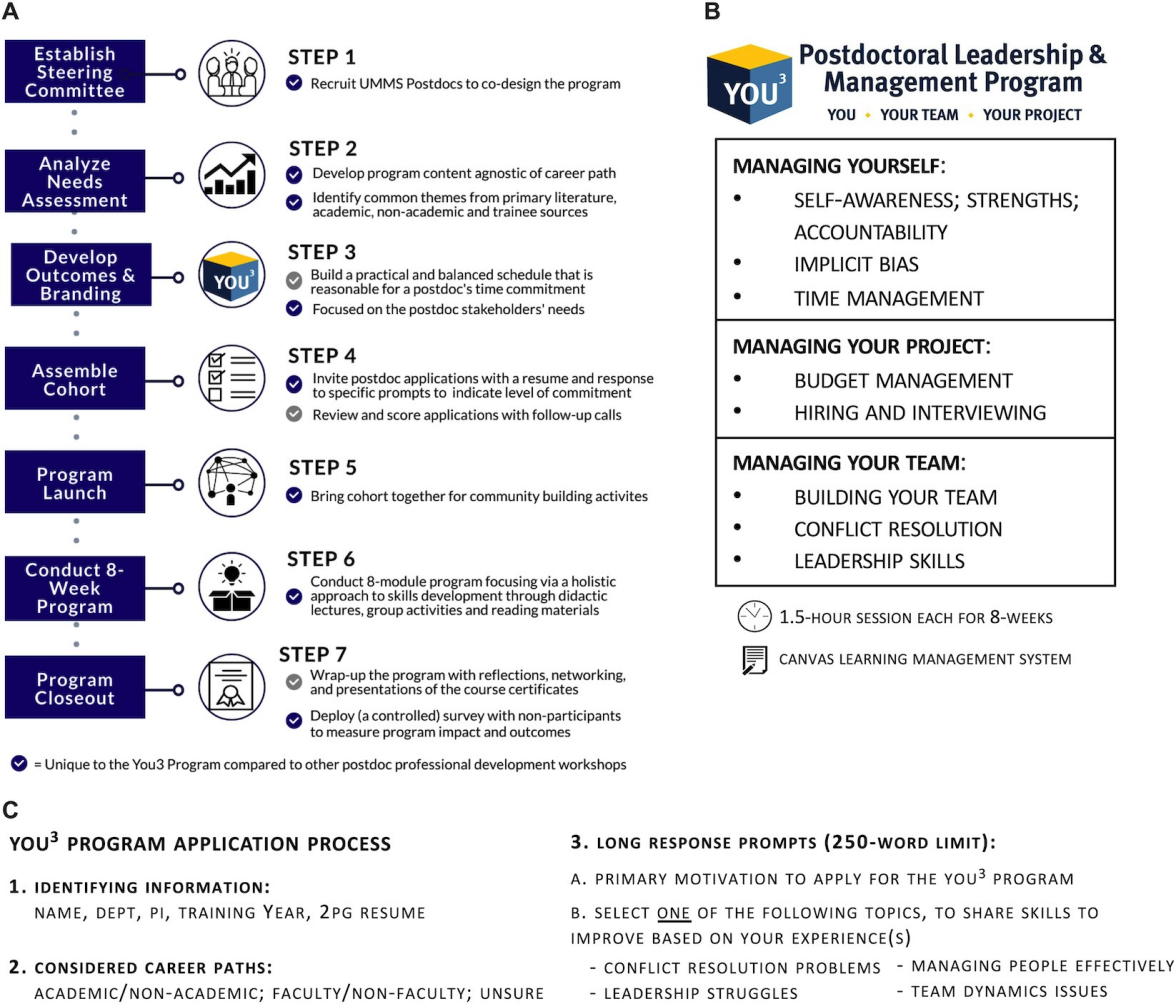
Overview of You^3^ program planning, framework, and application process. (A) Overview of the seven steps of planning, advertising, execution, and outcomes measurement of the You^3^ program. (B) Based on combined needs assessment and survey data, the You^3^ program content addressed 3 major themes: Managing Yourself, Managing Your Project, and Managing Your Team. Topics were then organized by theme, and each one was presented over the 8-week program in the order indicated in the table. (C) Summary of application process form.

The needs assessment above identified points for discussion and led to the development of the program methodology (Fig 2A). This was based on literature- and data-based discussions among the steering committee, which, importantly, consisted of four postdoctoral fellows who co-designed the curriculum. The program synthesizes the common needs into eight major topics that met the criteria of priorities for postdocs in a career-agnostic manner. These topics were organized into three themes: Managing Yourself, Managing Your Project, and Managing Your Team (Fig 2B). This process also resulted in branding the program as *You*^*3*^: *Leadership and Management Program for Postdocs*: You, Your Team, Your Project (Fig 2B) (ogps.med.umich.edu/you3).

**Fig 3:**
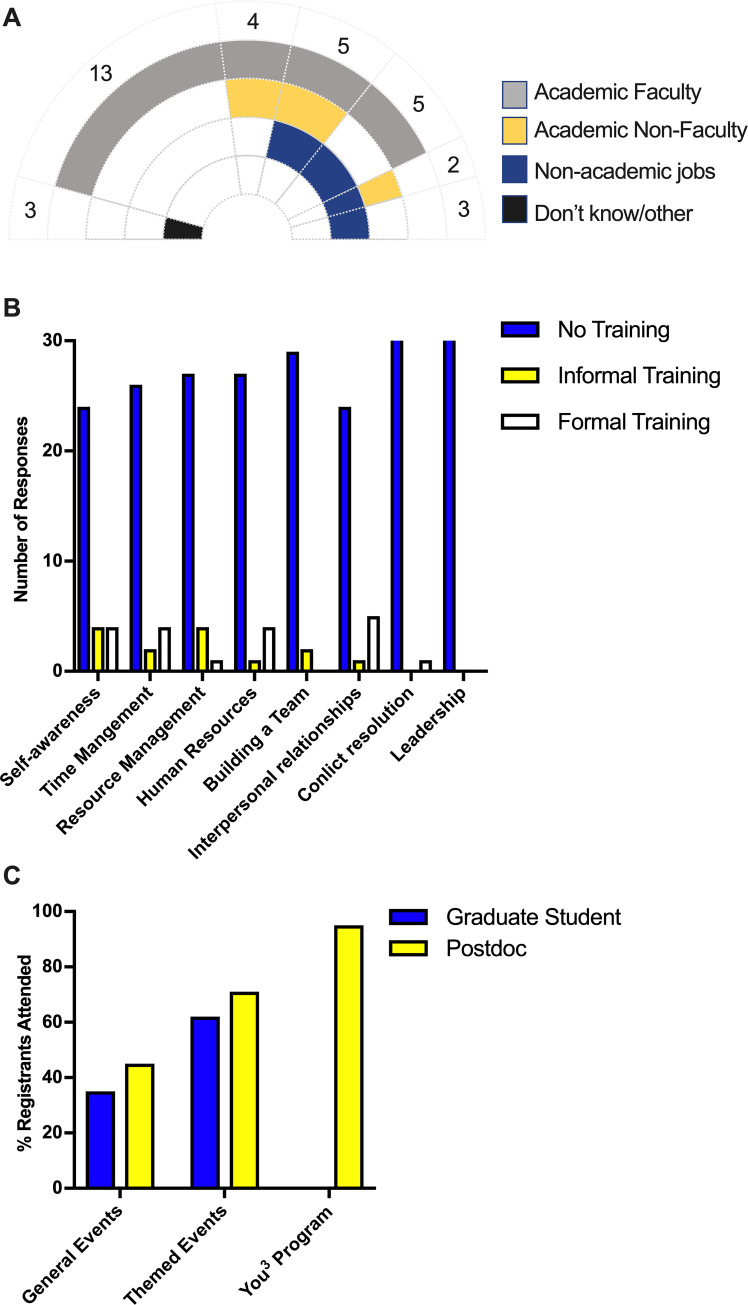
You^3^ participants have diverse career interests but little formal training in professional skills, and they display high commitment to the You^3^ program. (A) Applicants were asked to select one or more career paths they were currently interested in from the broad categories of Academic faculty, Academic non-faculty, Non-academic jobs, and Don’t-Know/Other. 45% of applicants had more than one future career interest. (B) A pre-program anonymous survey asked the You^3^ participants to report any training they had received in Self-awareness, time management, resource management, human resources, building a team, and interpersonal relationships. Their responses were categorized into formal training (led by an instructor with engagement activities, i.e. workshop, class), informal training (purely self-driven, i.e. online webinar, reading, etc.), and no training prior to enrollment. (C) Attendance data was collected from PhD students and postdocs from themed and non-themed events organized by our office. The percent registered attendance is compared to the average attendance percentage of the You^3^ participants during the program.

We formally launched the inaugural You^3^ program in September 2019 using email marketing to all postdoctoral fellows in UMMS and posted flyers across our research buildings. An open invitation intentionally queried diverse motivations for engaging in this program as evident in the application prompts and selection protocol (Fig 2C and S1 Fig). Applicants were required to submit a two-page resume and respond to one of four simple prompts on the topics of conflict resolution, interpersonal and team-work experiences, leadership, and project management (Fig 2C). The application material was used during the selection process to gauge their interest and commitment. We also gathered data on applicants’ current career plans and prior training in the program’s core competencies. As seen in Fig 3A, out of 35 applicants, 16 were certain about one career path, whereas the remaining 16 chose more than one potential path, 2 were unsure, and 1 applicant filled in a write-in response in the ‘other’ category. A pre-program anonymous survey indicated that the majority of applicants also reported they had little to no formal training in the skills covered in the You^3^ program, further highlighting the gap in professional training postdocs receive (Fig 3B). Finally, these 35 applicants represented 13 different clinical and basic science departments at UMMS. After reviewing the applications, we conducted phone interviews for a small subset to confirm motivation and commitment, which led to the committee inviting 33 postdoctoral fellows to participate with 32 ultimately matriculating into the You^3^ program. Fig 2A illustrates the overall framework for designing and implementing the You^3^ program, which can be adapted as a blueprint by other institutions.

The program consisted of one 90-minute meeting per week for eight consecutive weeks. In addition, there was a welcome event the week prior to the first session and a closing reflection ceremony the week after the last session. Each 90-minute in-class session was led by a subject matter expert external or internal to our institution. The external experts included an academic leadership coach (led four sessions) and a seasoned career consultant (led two sessions). The internal experts included an organizational learning specialist, who led the time management session, and two different unit-level senior administrative leaders, who co-facilitated the session on budgets and finances. The sessions, as such, were a mix of didactic instruction, group activities to enact or respond to scenarios, reflect on, and discuss pertinent topics. Pre-work, outside of class, included short readings and occasional self-assessment material. To further instill structure and weekly learning objectives of the You^3^ program, the program content and calendar reminders were organized on Canvas, a Learning Management System used for courses at our institute. Finally, to facilitate honest conversations and maintain a safe space, there was a community agreement on maintaining confidentiality outside of the class.

Attendance was required for participants to receive a certificate of completion. This not only encouraged strong commitment to the program (Fig 3C), but it also enabled sequential learning. We permitted pre-approved absences and excused attendance under reasonable circumstances. A small number of participants who could not physically be on campus chose the option of watching the program live stream on a video-conferencing platform. You^3^ participants had significantly higher attendance percentages overall compared to other workshops held by our unit, the Office of Graduate and Postdoctoral Studies. Specifically, *ad hoc* professional development workshops during the same period had a postdoc attendance of 45% of those who submitted an RSVP, and themed professional development workshops was at 71%. Conversely, the You^3^ program consistently had an attendance record of 95% (Fig 3C).

To measure the impact of the You^3^ program on participants, we decided not to rely on instructor evaluations due to their known biases [15] and inadequacies [16] or on conventional satisfaction surveys that do not address learning outcomes. Instead, at the end of our program, we surveyed You^3^ participants as well as non-participating postdocs as a control group to determine self-reported perceptions of a) current knowledge and b) growth during the program time period on each of the eight program topics (refer to S1 Appendix for the survey questions). We obtained twice the number of control group respondents as compared to the participants. Obtaining higher numbers in the control group was critical, as this enabled stratification between postdocs that have participated in any professional development programming (regardless of subject matter) at the University of Michigan and those that did not participate in relevant programming during the same months when the You^3^ program was held.

We used two sets of questions from our survey instrument to measure self-perceptions of a) end-of-program knowledge and b) growth in each module during the duration of the program. This instrument was administered immediately after the program completion to compare knowledge and growth of participants as compared to nonparticipants. The Cronbach’s alpha score was determined for the two primary question blocks stratified by cases and controls (Table 1). These data indicated that our instrument was highly internally reliable.

**Table 1:** Survey instrument reliability analysis.

Question Set ↓	All	Participants	Control
Respondent Type→	*Cronbach’s alpha Score (95% CI range)*	*Cronbach’s alpha Score (95% CI range)*	*Cronbach’s alpha Score (95% CI range)*
**Growth Question Set**	0.95 (0.93–0.96)	0.84 (0.73–0.94)	0.9 (0.86–0.94)
**Current Knowledge Question Set**	0.83 (0.77–0.88)	0.83 (0.72–0.93)	0.78 (0.69–0.87)

In order to assess survey reliability, Cronbach’s alpha was calculated across relevant sets of questions used to measure current knowledge and growth during program duration. CI = confidence interval.

**Fig 4:**
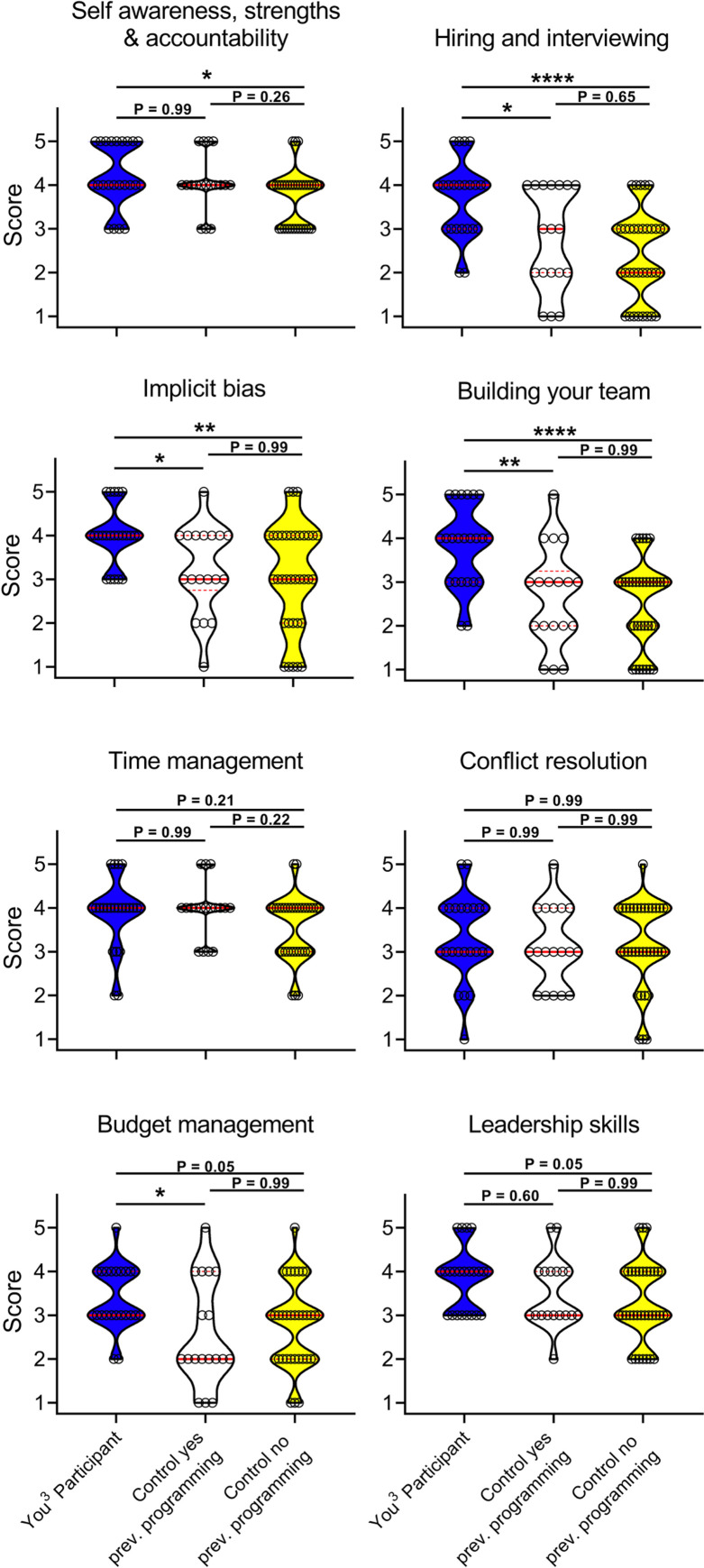
Cross-sectional assessment reveals that You^3^ participants have a higher perception of knowledge than controls across You^3^ program modules. You^3^ participants and controls self-assessed their current knowledge (high = 5, low = 1) for each of the eight You^3^ program modules. Controls were then stratified by previous participation in any career development programming. Current knowledge scores for each module were compared between You^3^ participants (blue, n = 23), controls who had participated in any career development programming (white, n = 18), and controls who had not participated in any career development programming (yellow, n = 35), median (solid red line) and interquartile range (dotted red line) displayed (*P < 0.05, **P < 0.005, ****P < 0.00005, Kruskal-Wallis test followed by Dunn’s posthoc test).

You^3^ participants self-reported greater knowledge at the end of the program on the topics of Hiring and Interviewing, Implicit Bias, Building Your Team, and Budget Management as compared to control groups that had received other professional development programming (Fig 4). Additionally, compared to the no-other-program controls, You^3^ participants reported greater knowledge in all areas with the exception of time management and conflict resolution (Fig 4). While we cannot quantitatively measure participants’ practical skills in these areas via traditional exams or quizzes, our results indicate the high value You^3^ participants experienced from this program.

**Fig 5:**
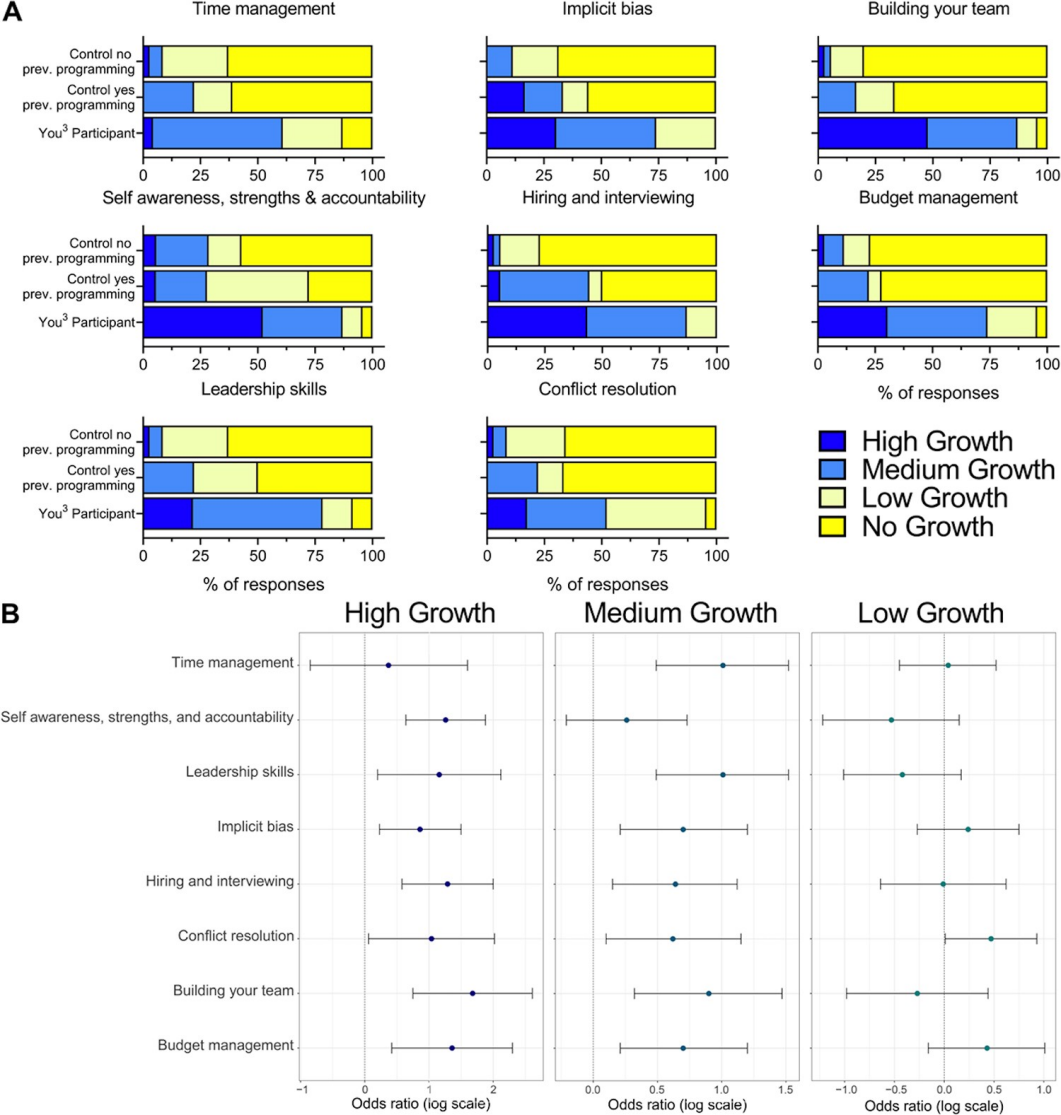
You^3^ participants indicate higher perception of growth across You^3^ program modules compared to controls. You^3^ participants and controls self-assessed their growth (4 = High Growth, 3 = Medium Growth, 2 = Low Growth, 1 = No Growth) over the You^3^ program timeframe for each of the eight You^3^ program modules. Controls were then stratified by previous participation in any career development programming. (A) Growth in each module was compared between You^3^ participants (n = 23), controls who had participated in any career development programming (n = 18), and controls who had not participated in any career development programming (n = 35). (B) Odds ratios for “High Growth,” “Medium Growth,” and “Low Growth” were calculated comparing You^3^ participants (n = 23) to all controls (n = 53) for each of the eight You^3^ program modules (95% confidence intervals displayed).

To determine if the relationship between comparatively independent, higher self-reported knowledge is associated with participation in the You^3^ program, we also surveyed You^3^ participants and controls on their self-reported growth in each of these skills over the timespan of our program. You^3^ participants reported the highest growth across all skills (Fig 5A). Associative analysis revealed that You^3^ participants had significantly higher odds of reported high and medium growth in 7 of 8 metrics, and low growth in 1 of 8 metrics (Fig 5B). These data were stratified into “High,” “Medium,” and “Low” to determine the strength of association between participation in the You^3^ program (exposure) and self-reported growth (outcome). This contrasts with a binary “Growth/No growth” stratification of the data, which may mask this association by aggregation of “Low growth” and “High growth” responses. These data indicate that individuals that participated in the You^3^ program were more likely to report growth than non-participants in all You^3^ modules except Conflict resolution; therefore, participation in the You^3^ program was highly associated with perceived growth in the topics covered by the You^3^ program. This highlights the value of the You^3^ program as participants feel they have developed greater growth and knowledge in the surveyed topics compared to the control groups.

To determine if self-reported knowledge in one topic correlated with knowledge in any of the other seven topics or with years in postdoctoral training (median years in postdoctoral training = 1 year, range = 0–5 years), we also performed Pearson’s correlation analyses. We did not find consistent patterns from this analysis when measuring self-reported knowledge correlations for You^3^ participants and previous programming controls. However, we did consistently find statistically significant correlations between all of the eight-knowledge metrics in the no previous programming control group (S2A Fig). These correlations were generally driven by a paucity of self-reported knowledge across our topics, indicating a baseline insufficiency in postdoctoral training related to professional skills. A similar trend was observed when the same correlation analysis was conducted between the self-reported growth metrics, and growth was independent of years in postdoctoral training (S2B Fig). These data indicate that participation in transferable skills development programming is associated with positive growth. Moreover, we posit that the You^3^ program is more effective at encouraging positive growth than *ad lib* engagement with transferable skills development programming.

## Discussion

Our study describes a novel program that implements a cohort-based structured curriculum and measures confidence via self-perceived knowledge and growth in key transferable skills in this cohort compared to a control cohort of non-participants. We show that participation in our program resulted in a measurable increase in the participants’ self-reported knowledge and growth in multiple non-technical areas, compared to control non-participants. Interestingly, the increase was independent of time spent as a postdoc, indicating that experience and time alone was not enough to develop skills, and that active programming was necessary.

A cohort-based structured program, which is uncommon for professional skill development activities for postdocs, likely contributed to the success of the program in several ways. First, it clearly improved attendance. We saw an unprecedented (95%) continuous attendance in our program, which was substantially higher than the ~45% attendance we have seen in the ad hoc programs we have offered. Second, the 8-week, structured program could have mitigated a key hurdle in postdoc engagement in professional skill building activities. The short time frame and sporadic offerings of traditional ad hoc workshops, makes it difficult for postdocs to identify, determine value, and attend relevant programs that productively assist them in transitioning to their next stage [1, 9, 17]. Providing a structure and sequence likely lessened the above hurdle. Third, the cohort-based discussions provided an opportunity to recognize, valuate, and address professional challenges that participants collectively found most pressing. Fourth, the cohort helped build community among postdocs for continued interactions, even after the program ended, similar to what is seen in graduate students. These inferences are consistent with previous studies which suggest that cohort-based facilitates better experiences, such as in-depth discussions of sensitive topics [18, 19].

Another key innovation that helped spur engagement was the extent of input from and involvement of four current postdocs, representatives of the target population, in designing the program. This is a notable difference between You^3^ and most previous programs. In addition to providing boots-on-the-ground perspectives, the postdocs who helped develop the program had the added benefit of growing as leaders and strategic thinkers. These benefits are consistent with previous reports where engaging college students early has been valuable in developing teaching methods and planning curricula [20].

The self-reported confidence and growth we used to assess outcomes, based on the scientific method, requires careful consideration. Traditionally, educational and professional development programs rely on satisfaction surveys and/or instructor ratings. While these assessment methods are useful to provide general overviews of course effectiveness, they are limited by issues such as potential biases and lack of depth [21, 22]. Attempts to assess outcomes in more quantitative ways have been challenging, as programs such as ours typically do not (and should not) use grades as a measurement of learning. The controlled assessments, comparing participants’ perceived knowledge and growth to that of non-participants in the same time frame, was developed based on a method used in medical settings such as clinical trials. Self-perceived knowledge and growth reporting which convey awareness and confidence, but do not directly test competence, are components of lifelong learning [23–25]. Similarly, awareness contributes to conceptual understanding by allowing learners to review and reconstruct their understanding of a skill. Transferable skills have traditionally been difficult to directly assess in a controlled and quantitative manner, and our method of self-rating growth could serve as a reliable proxy.

Because self-assessment and awareness form a significant part of the assessments, it is possible that the assessments were influenced by participants’ awareness of what they did not know earlier. Additionally, there is a possibility that participants self-reported growth reflects familiarity with program terminology rather than growth in understanding. The case-control design of the evaluation of this program had the added benefit of including You^3^ non-participants that participated in alternative career development programming over the same time frame. These data show that You^3^ participants exhibited more self-reported growth than non-participants that had participated in alternative career development programming. Furthermore, this latter control group reported more growth in several You^3^-specific modules compared to non-participants that did not engage in career development programming. This suggests that self-reported growth reflects awareness rather than mere terminological understanding, as it is unlikely that the specific terminology used in the You^3^ program was exactly reflected in alternative career development programming. This inference is especially likely given that You^3^ program instructors and content differed from that of alternative career development programming. Therefore, the growth reported by these individuals likely reflects higher awareness of You^3^-specific competencies. Nonetheless, continued interrogation of acquisition and application of transferrable competencies is valuable in longitudinal analyses of such programs.

We also considered whether erroneous superiority or the Dunning-Kruger effect [26] could affect our results. The control group’s self-awareness was likely more variable than that of the You^3^ participants, because the control group’s previous participation in relevant training and the intensity of training was unpredictable, while the You^3^ participants, as a cohort, uniformly went through the same set of workshops meant to increase awareness and confidence. Therefore, the responses of the control group could be less calibrated and uniform than that of the You^3^ participants. This variability was partly mitigated by having twice the number of respondents in the control group. This further enabled stratification of the control population into groups: those that have participated in any professional development programming (regardless of subject matter) at the University of Michigan and those that did not participate in relevant programming during the same months. Compared to both control groups, You^3^ participants reported higher growth in relevant competencies. In any case, a Dunning-Kruger effect driven by decreased self-awareness in control groups would lead to erroneously increased confidence in the control groups, therefore biasing our results to a null finding. The fact that we are seeing the opposite—an increased confidence in You^3^ participants—despite this potential confounding effect, underscores the effectiveness of the program.

The self-assessment method of measuring outcomes, while it has clear strengths as outlined above, also has limitations that need to be considered while interpreting the data. Although the self-perceived growth in knowledge that we measure is correlated with learning, we do not directly evaluate competence by external objective measurements such as mentor interviews. Within the scope of our study, we opted not to interview postdoc mentors because of the variability in the extent of mentor buy-in for participation in such programs and the disparities in levels of interactions between mentors and postdocs, neither of which we could control for. Further, our participants opted to apply to this program, and therefore could have been more interested or aware than our control group to start with. However, the participants’ initial levels of awareness or confidence are unlikely to bias our results. That is, even if we started with a group of individuals that had a higher level of awareness in the beginning, their self-perceived knowledge grew more than that of the control group, which could only widen the difference at the end. Another consideration is that our control group included individuals who participated in other professional development programing. The structure and content of these programs, which we could not record for individual responders, could have differed by varying degrees compared to the respective sessions in You^3^. However, we observed a difference in growth in our participant group even compared with this broad group, indicating that overall, the You^3^ format improves awareness and confidence in these topic areas over time.

The outcomes and the success of the inaugural offering as described in this manuscript has informed us to continue this program, while making it stronger for future offerings. But due to the sudden changes to in-person gatherings and due to significant budget cuts, our emphasis for the next cohort was to optimize the program for virtual and hybrid formats during the COVID-19 pandemic. Albeit virtual, based on feedback from the 2019 cohort, we implemented a greater number of activities and assignments to be completed during and outside of the session. While we are still collecting feedback from these changes, our goal was to increase experiential learning and build community among postdocs who often suffer from isolation and loneliness [27, 28]. Our 2020–21 experience, along with another recent study our office conducted, unearthed several advantages of virtual professional development programming, prompting us to likely keep a virtual component in some form [29].

An aspect of the program that we plan to develop and build on is long-term tracking of the postdocs who have participated in the program. We anticipate gathering data on not only their professional paths but also intend to set up mechanisms to best understand how this program set the foundation for lifelong learning in critical areas such as emotional intelligence [30, 31] and related interpersonal skills. While we are in the process of building a robust longitudinal survey to be deployed a few months after the COVID-19 pandemic recedes and the professional development training space returns to some level of normalcy, we are beginning the process of contacting our program alumni. Our first cohort of participants were very responsive (n = 19) to a check-in survey on how they are using skills from the You^3^ program in their professional journey so far. Data from this quick survey indicate that 57% of responding alumni have transitioned to a new a position or have started a leadership role in their current position and 26% will soon be transitioning into a new career or leadership role. While we cannot directly attribute any career transitions to participation in our program, the respondents pointed to several of the skills covered in the You^3^ modules as being important in their career preparation. This indicated that since the program completion, they have continued to actively improve their skills covered in the You^3^ program. The skills that the alumni respondents are continuing to improve include, but not limited to, self-awareness, conflict-resolution, implicit-bias, time management, and team management. These preliminary data inform us in the building of a longitudinal tracking survey instrument. Such long-term data, along with information on how other institutions are implementing similar programs, will strengthen our overall assessment. Using this iterative approach, we aim to optimize the program to its most effective format in the coming years.

The You^3^ program is a novel, scalable, and rigorous framework that can be adapted by any institution to expand the foundation of postdoctoral training. Our structured program and scientific assessment of outcomes helps minimize the variabilities and inequities in experiences and consequences prevalent in the current apprenticeship-based training models. For example, postdocs don’t have equitable time or guidance to access professional skill building and career development, despite recommendations from national bodies like the National Postdoc Association and relevant studies [2, 32]. A subset of postdocs who are independently funded, such as by the NIH’s K or F award systems, require professional development plans to be submitted in the proposal and followed through. On the contrary, postdocs who are not independently funded don’t have any mechanism to consider professional development. Structured programs such as You^3^, developed via postdoc-centric guiding principles, mitigate such inequities by providing access to professional development in a structured manner to all postdocs regardless of funding status. Our program is an important benefit for international postdocs, who often don’t get the opportunity to craft plans via funding proposals, as they are often ineligible for independent funding.

Finally, based on our experience, we believe You^3^ can be adapted easily to institutional needs, including hybrid training models combining in-person and virtual formats. We anticipate that newly funded initiatives from the NIH, such as The Postdoc Academy (postdocacademy.org), will provide complementary and supplementary resources to make broad implementation feasible within the budgets of most institutions.

## Supporting information

S1 Fig Application questionnaire. This application via Google forms was sent to all UMMS Postdocs to solicit participants.
(TIF)

S2 Fig Pearson’s correlation analysis of self-reported knowledge and growth. (A) Current knowledge scores for each module were correlated to all other modules and the number of years prior to 2019 in their postdoctoral fellowship, and Pearson correlation r and P values were calculated for You^3^ participants, controls who had participated in any career development programming, and controls who had not participated in any career development programming. (B) Growth scores for each module were correlated to all other modules, and Pearson correlation r and P values were calculated for You^3^ Participants, controls who had participated in any career development programming, and controls who had not participated in any career development programming. R values are displayed, and P values are summarized in red (*P < 0.05, **P < 0.005, ****P < 0.0005).
(TIF)

S1 Appendix Survey questions. Survey questions for You^3^ participants to measure their self-reported knowledge and growth around the eight modules after completing the program. Survey questions for You^3^ non- participating UMMS postdoctoral fellows. All were asked to report their knowledge and growth on the same metrics and time frame of the You^3^ program.
(PDF)

S1 Dataset Application, pre-course, and attendance. (XLSX)

S2 Dataset Outcomes survey and data key. (XLSX)
