# Supplementary figures and images for: A structured professional development curriculum for postdoctoral fellows leads to recognized knowledge growth
Source: PLoS One. 2021 Nov 22;16(11):e0260212. doi: 10.1371/journal.pone.0260212 (PMC8608334; doi:10.1371/journal.pone.0260212)

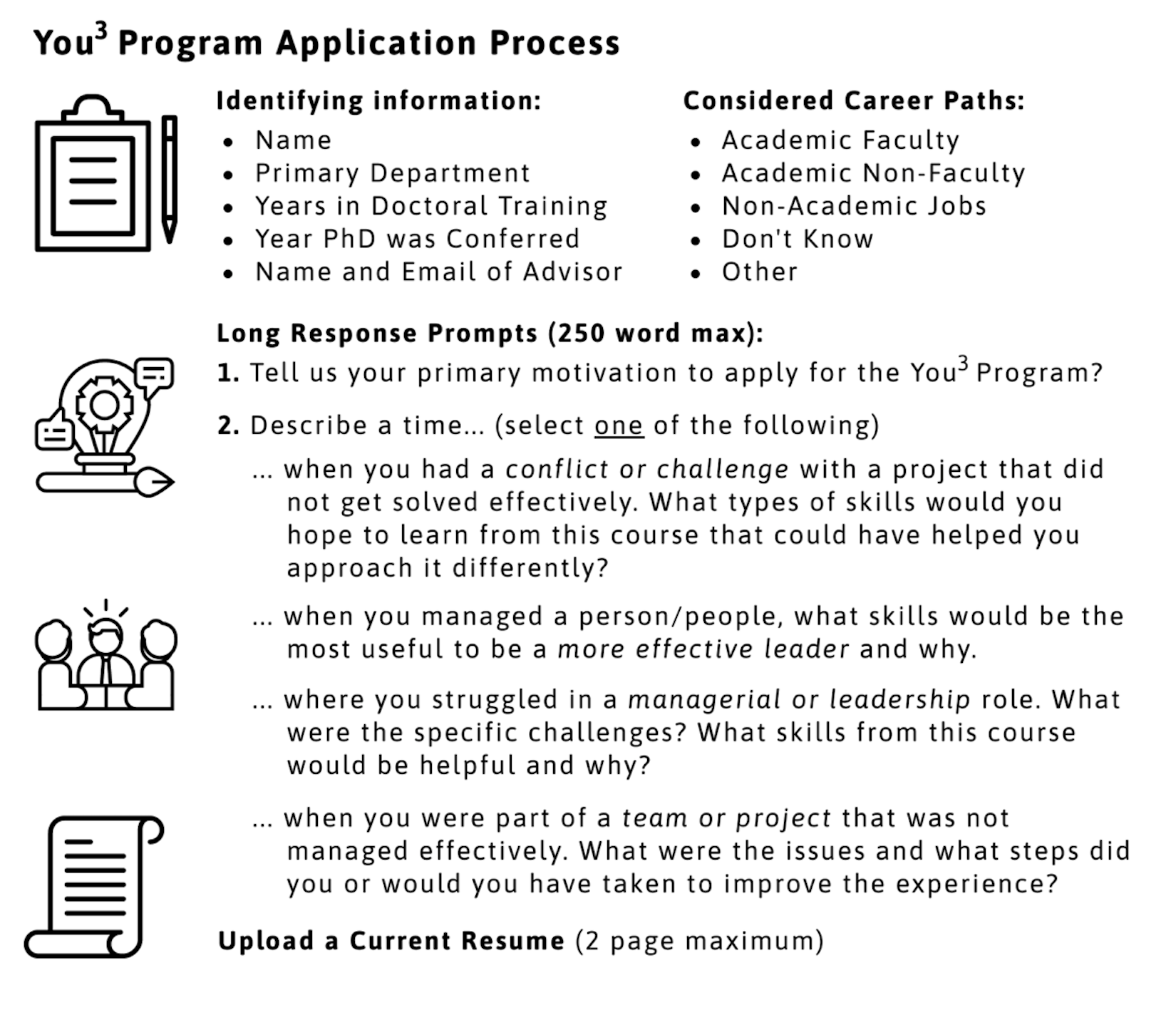

Supplement: S1 Fig — This application via Google forms was sent to all UMMS Postdocs to solicit participants. (TIF) [file pone.0260212.s001.tif]

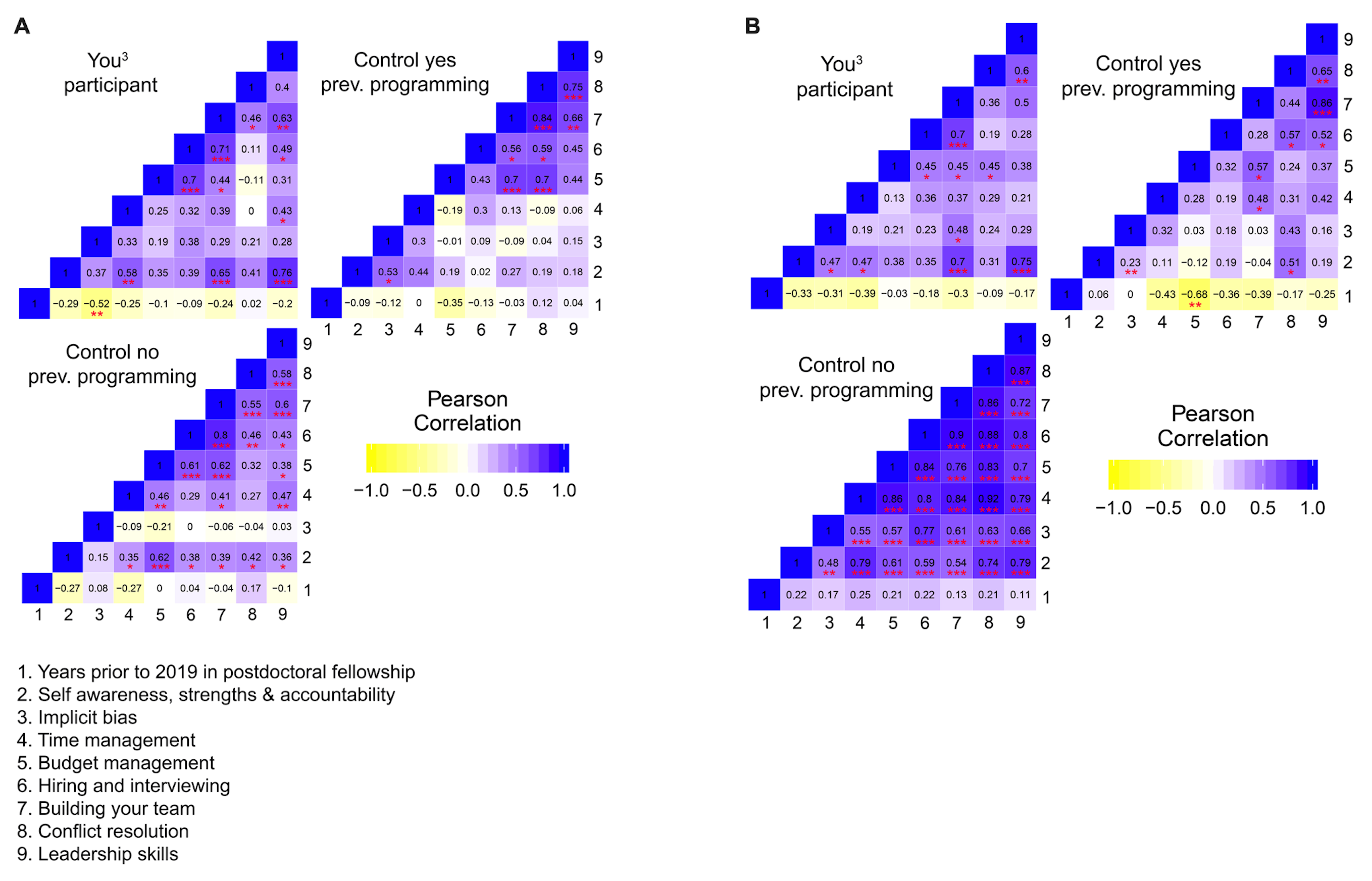

Supplement: S2 Fig — (A) Current knowledge scores for each module were correlated to all other modules and the number of years prior to 2019 in their postdoctoral fellowship, and Pearson correlation r and P values were calculated for You3 participants, controls who had participated in any career development programming, and controls who had not participated in any career development programming. (B) Growth scores for each module were correlated to all other modules, and Pearson correlation r and P values were calculated for You3 Participants, controls who had participated in any career development programming, and controls who had not participated in any career development programming. R values are displayed, and P values are summarized in red (*P < 0.05, **P < 0.005, ****P < 0.0005). (TIF) [file pone.0260212.s002.tif]
