# Supplementary material for: A structured professional development curriculum for postdoctoral fellows leads to recognized knowledge growth
Source: PLoS One. 2021 Nov 22;16(11):e0260212. doi: 10.1371/journal.pone.0260212 (PMC8608334; doi:10.1371/journal.pone.0260212)
Supplement: S1 Appendix — Survey questions for You3 participants to measure their self-reported knowledge and growth around the eight modules after completing the program. Survey questions for You3 non- participating UMMS postdoctoral fellows. All were asked to report their knowledge and growth on the same metrics and time frame of the You3 program. (PDF) [file pone.0260212.s003.pdf]

Block 3

We're inviting you to participate in a research study to enhance current and future professional development programs for postdoctoral fellows at the University of Michigan. Participation is completely voluntary and response to any survey questions indicates your consent. Data collection is anonymous, however; once the survey is initiated (answering a question), your data cannot be removed. There are no negative consequences to participation, whatever you decide. If you have any questions or concerns, please contact Shoba Subramanian at shobas@umich.eduor 734-615-6511.

Block 1

Select the year when you started as a postdoc at the University of Michigan

- 2014
- 2015

- ○ 2016
- ○ 2017
- ○ 2018
- ○ 2019

Measuring Participants' Knowledge and Growth for Each of the You3 Topics

	Rate on a scale of 1-5 your ****current** end-of-program** knowledge in each of the You3 topic modules (1: very low; 3: medium; 5: very high)					Document any **increase in knowledge as a result the You3 program** participation. (Calibrate your baseline to knowledge before program started)			
	1	2	3	4	5	No Growth	Low Growth	Medium Growth	High Growth
Self awareness, strengths & accountability	○	○	○	○	○	○	○	○	○
Implicit bias	○	○	○	○	○	○	○	○	○
Time management	○	○	○	○	○	○	○	○	○
Budget management	○	○	○	○	○	○	○	○	○
Hiring, Interviewing	○	○	○	○	○	○	○	○	○
Building your team	○	○	○	○	○	○	○	○	○
Conflict resolution	○	○	○	○	○	○	○	○	○

	Rate on a scale of 1-5 your ****current** end-of-program** knowledge in each of the You3 topic modules (1: very low; 3: medium; 5: very high)					Document any **increase in knowledge as a result the You3 program** participation. (Calibrate your baseline to knowledge before program started)			
	1	2	3	4	5	No Growth	Low Growth	Medium Growth	High Growth
Leadership skills	○	○	○	○	○	○	○	○	○

Measuring applicability of knowledge gained from You3 program participation. The application can be for a current or potential area of concern and can be in current postdoc position or in a career after your postdoc training.

	I plan to use the below skills & knowledge learned in You3 in a future professional space (current position or new career).		Provide 1-2 examples of where you will/could use knowledge from this module. If you answered "no", then say n/a. Example: Lab mentoring, ongoing collaboration, potential conflict, planning job applications, etc.
	Yes	No	Type 1-2 examples here
Self awareness, strengths & accountability	○	○	[ ]
Implicit bias	○	○	[ ]
Time management	○	○	[ ]
Budget management	○	○	[ ]

	I plan to use the below skills & knowledge learned in You3 in a future professional space (current position or new career).		Provide 1-2 examples of where you will/could use knowledge from this module. If you answered "no", then say n/a. Example: Lab mentoring, ongoing collaboration, potential conflict, planning job applications, etc.
	Yes	No	Type 1-2 examples here
Hiring, Interviewing	○	○	
Building your team	○	○	
Conflict resolution	○	○	
Leadership skills	○	○	

List any other workshop** you participated in between Sept and Nov 2019. Note: A workshop (in this context) is defined as a 1-hour or longer in-person program on a professional development topic led by an expert.

You3 Program Evaluation

Overall, how satisfied or dissatisfied were you with the You3 Program?

- Extremely satisfied
- Moderately satisfied
- Slightly satisfied
- Neither satisfied nor dissatisfied
- Slightly dissatisfied
- Moderately dissatisfied
- Extremely dissatisfied

How likely are you to recommend this course to a friend or classmate?

Not at all likely — Extremely likely

0 1 2 3 4 5 6 7 8 9 10

How much did you learn from this course?

- A great deal
- A lot
- A moderate amount
- A little
- Nothing at all

How reasonable or unreasonable was the time allocated for this course?

- Extremely reasonable
- Moderately reasonable
- Slightly reasonable
- Neither reasonable nor unreasonable
- Slightly unreasonable
- Moderately unreasonable
- Extremely unreasonable

How knowledgeable was the instructor of the material presented in this course?

- Extremely knowledgeable
- Very knowledgeable
- Moderately knowledgeable
- Slightly knowledgeable
- Not knowledgeable at all

How well did this course meet your expectations?

- Extremely well
- Very well

- ○ Moderately well
- ○ Slightly well
- ○ Not well at all

What did you like most about this program?

What did you like least about this program?

How could this program be improved?

Block 3

Thank you for participating in this inaugural program and for filling out the survey!

Powered by Qualtrics

UNIVERSITY OF MICHIGAN

Purpose of Survey

We're inviting you to participate in a research study to enhance current and future professional development programs for postdoctoral fellows at the University of Michigan. Participation is completely voluntary and response to any survey questions indicates your consent. Data collection is anonymous, however; once the survey is initiated(answering a question), your data cannot be removed. There are no negative consequences to participation, whatever you decide. If you have any questions or concerns, please contact Shoba Subramanian at shobas@umich.eduor 734-615-6511.

Participation in You3

Have you heard of the You3 Leadership & Management Program for Postdocs at the University of Michigan Medical School?

- ○ Yes
- ○ Maybe

- No

Did you participate in the OGPS You3 Program - Sept-Nov 2019?

- Yes
- No

Select the year when you started as a postdoc at the University of Michigan

- 2014
- 2015
- 2016
- 2016
- 2017
- 2018
- 2019

Measure You3 Topic Knowledge & Growth

Measuring Knowledge and Growth for Leadership & Management (as they apply in a professional work place).

	Rate on a scale of 1-5 your ****current** knowledge in each of the topics** (1: very low; 3: medium; 5: very high)					Document any **increase in knowledge (within the last 3 months)** based on participation in any recent workshops			
	1	2	3	4	5	No Growth	Low Growth	Medium Growth	High Growth
Self awareness, strengths & accountability	○	○	○	○	○	○	○	○	○
Implicit bias	○	○	○	○	○	○	○	○	○
Time management	○	○	○	○	○	○	○	○	○
Budget management	○	○	○	○	○	○	○	○	○
Hiring, Interviewing	○	○	○	○	○	○	○	○	○
Building your team	○	○	○	○	○	○	○	○	○
Conflict resolution	○	○	○	○	○	○	○	○	○
Leadership skills	○	○	○	○	○	○	○	○	○

List any workshops that you participated in between Sept and Nov 2019. Note: A workshop (in this context) is defined as a 1-hour or longer in-person program on a professional development topic led by an expert.

You3 Program Evaluation

If offered next year, will you apply to participate in the You3 Postdoc Leadership and Management Program?
You3 Program Link

- Extremely likely
- Somewhat likely
- Neither likely nor unlikely
- Somewhat unlikely
- Extremely unlikely

Optional: If you are unable to participate in You3, what are the barriers to this?

Optional: What other professional development workshops will you attend, if offered? Please list topics.

Block 4

Thank you for clicking on this survey, since you participated in You3, please respond to the survey posted via the Canvas site tailored for program participants

Thank you for filling out this survey!

Powered by Qualtrics
